# Third-generation sequencing reveals the spatial variation of microbial composition of airborne bacteria in an intensive dairy farm

**DOI:** 10.3389/fmicb.2025.1688472

**Published:** 2025-12-15

**Authors:** Qianqian Zhang, Luyu Ding, Xueying Xie, Lin Ru, Qifeng Li, Chunxia Yao, Johann Solkner, Ruixiang Jiang

**Affiliations:** 1 School of Chemistry and Biological Engineering, University of Science and Technology Beijing, Beijing, China; 2 Information Technology Research Center, Beijing Academy of Agriculture and Forestry Sciences, Beijing, China; 3 College of Civil Engineering and Water Conservancy, Heilongjiang Bayi Agricultural University, Daqing, China; 4 National Innovation Center of Digital Technology in Animal Husbandry, Beijing, China; 5 Institute of Livestock Sciences, University of Natural Resources and Life Sciences, Vienna, Austria

**Keywords:** third generation sequencing, air microbial communities, phylogenetic tree, intensive dairy farming, risk of diseases

## Abstract

**Introduction:**

The intensification of livestock farming has led to increased bacterial bioaerosol emissions, posing potential health risks to both animals and humans. This study aimed to investigate the bacterial community composition, abundance, diversity, and variation in different functional zones of cattle farms to assess their impact on public health and environmental quality.

**Methods:**

We employed third-generation sequencing on the PacBio platform to analyze 16S ribosomal RNA (rRNA) sequences from air samples, identifying a diverse range of bacterial phyla, including *Proteobacteria*, *Firmicutes*, *Verrucomicrobia*, *Bacteroidetes*, *Fusobacteria*, *Actinobacteria*, *Deinococcus-Thermus*, *Cyanobacteria*, and *Acidobacteria*. The phylogenetic tree was built using the microbiome abundance of these samples.

**Results:**

Notably, Firmicutes and Proteobacteria were predominantly enriched in the samples, with genera such as *Staphylococcus*, *Acinetobacter*, *Enterococcus*, and *Bacillus*, and the family *Enterobacteriaceae*, which was unknown, being particularly abundant. These bacteria are known to be associated with various infections and chronic diseases. Correlation and canonical correspondence analysis (CCA) revealed that environmental factors, particularly ultraviolet (UV) radiation and global horizontal irradiance (GHI), significantly influence microbial species distribution, with *R*^2^ values of 0.774 (*p* < 0.05) and 0.769 (*p* < 0.05), respectively. We further calculated the alpha and beta diversity of microbiome in these samples and observed that fermenting manure (F1), fresh manure (X2), and piled-up manure after fermentation (D3) samples have the highest alpha diversity, while PC1 from beta diversity, that is, weighted principal coordinates analysis (PCoA), explained 32.66% of the variance in the data. Interestingly, the relative abundance of the *Kocuria* genus was significantly different between the waste management area (FW) and the milking parlor (NT) (*t*-test, *p* < 0.013).

**Discussion:**

Our findings underscore the importance of understanding the complex microbial ecosystems in livestock farming environments and highlight the need for targeted interventions to mitigate public health risks associated with bacterial bioaerosols.

## 1 Introduction

Intensive livestock farming is an important source of bacterial bioaerosol emissions, and exposure to these emissions has drawn widespread public concern (Douglas et al., 2018; Gladding et al., 2020; Humbal et al., 2018). A systematic review conducted across 22 countries and regions investigated potential threats to respiratory health posed by microbial bioaerosol exposure. It has been shown that a substantially positive association between bioaerosol exposure and respiratory disease outcomes in targeted populations, and research has found that intensive animal farms are the sites with higher bacterial bioaerosol concentrations (Iqbal et al., 2024). However, other researchers have also shown that being born or raised on a farm is associated with less atopic sensitization than in the general population (Portengen et al., 2005; Elholm et al., 2013; Illi et al., 2012). Vestergaard et al. (2018) investigated the composition and diversity of airborne bacteria collected from pig stables and farmers’ homes using 16S ribosomal RNA (rRNA) sequences.

Compared to the controls (i.e., suburban homes), there was a higher abundance of bacterial groups potentially causing a protective effect against asthma and allergy, such as phyla *Firmicutes* and *Actinobacteria*, and the families *Prevotellaceae*, *Lachnospiraceae*, and *Ruminococcaceae* (Zhang et al., 2018), and *Peptostreptococcaceae* (Vestergaard et al., 2018), in pig stables and farmers’ homes. Meanwhile, air in pig stables and farmers’ homes with a higher abundance of bacterial groups caused negative health consequences, such as species within the genus *Staphylococcus* (Vestergaard et al., 2018). Thus, further analysis of the composition and diversity of airborne bacteria in livestock farming would improve our understanding of the role of airborne bacteria on the influence of public health, the risk of diseases in the environment, and provide valuable data for constructing air flow models to create a better livestock farming environment (Kim et al., 2009; Ruiz-Gil et al., 2020).

Compared to pigs and poultry, dairy cattle are typically free-ranging or housed in naturally ventilated buildings, which makes it more convenient to examine the formation and dispersion of airborne bacteria (Ding et al., 2024). The previous studies focused more on pathogenic microorganisms in dairy farms. For example, seasonal variations in the microbiota of milk, feces, bedding, and airborne dust have been studied to investigate the relationship between mastitis and the microbiota. Generally, there is spatial variation in the concentration of airborne bacteria across different functional zones in typical dairy farms (Ru et al., 2023). A comprehensive analysis of both the negative and protective effects of airborne bacteria across different functional zones would aid in the development of precise biosafety measures in dairy farms and deepen the public’s understanding of airborne bacteria (Amin et al., 2022; Martikainen et al., 2021; McEachran et al., 2015). Both the negative and protective effects of microbiomes are interesting to study in terms of their structures and sequences, as some microbiomes can protect the host in certain circumstances while being detrimental in others (Amin et al., 2022; Martikainen et al., 2021; McEachran et al., 2015).

The objectives of this study are (1) to investigate and compare the composition structure of air microbial communities in different functional areas of cattle farms, and analyze the types of beneficial and harmful bacteria; (2) to disentangle the evolutionary relationships between air microbial communities; (3) to analyze the environmental factors affecting the community composition of airborne bacteria and their interactions in dairy farm.

## 2 Materials and methods

### 2.1 Sample collection

Field measurements were conducted on a commercial dairy farm in Beichen District, Tianjin, China (39.25°N, 117.05°E) with a stock density of 3,000 cows, milked 3 times a day and fed 3 times a day. To examine the microbial composition of airborne bacteria in dairy farm, airborne bacteria were sampled by the Coriolis *μ* samplers (Bertin Technologies, France) at a height of 1.2 m (medium: 15 mL ultrapure water; duration: 10 min; flow rate: 300 L min^−1^) for further 16S rRNA gene amplicon sequencing, the detailed information please referring to Ru et al. (2023). The specific sampling height (1.2 m) was used based on the National Standard No. GB/T 3817–2020 stated that the height could be 1.2–1.6 m based on the height at which the cows breathe. There was one sampling site, and there were two types of machines sampling at the same time: (1) six-stage Anderson samplers (Tisch Environmental, Inc. [TEI] Corporation, United States); (2) Coriolis *μ* samplers (Bertin Technologies, France). To determine the concentration of airborne bacteria, samples were also collected using the six-stage Anderson samplers (Tisch Environmental, Inc. [TEI] Corporation, United States) for incubation and colony counting. Each sampling point was equipped with these two instruments, and the inner surfaces of the instruments were wiped and disinfected with 75% alcohol between the two samplings.

This study collected air samples from different functional zones of a typical large-scale dairy farm in Northern China and performed third-generation sequencing of the V1–V9 region of bacterial 16S rRNA genes in these samples. Air samples were collected in eight zones, three replicates per zone, according to the different functions and potential pollution levels in the dairy farm, resulting in a total of 24 air samples collected. Theoretically, the office area (BG) and feed processing area (SL) are less polluted, while the waste management area (FW) would be more polluted due to centralized storage and strip drying of manure. Cows are normally housed in a dairy barn (SN) with free access to outdoor open lots (YDC) and are transferred to the milking parlor (NT) for milking two or three times a day in the production area. Interfiled roads (IR) between barns are used for the daily operation of workers and vehicles. The separation roads (ZD) between different dairy production areas and feeding areas are also sampled in this study. Thus, air samples were collected at the background point (BJ) and at eight different points within the sites of BJ, SL, FW, SN, YDC, IR, ZD, and NT. The background sampling points were located in the open area upwind direction of the dairy farm, and the dairy farm was more than 500 m away. To prevent the field production activities from contaminating the sampling (Ru et al., 2023), samples were collected at 11:30 ~ 12:30 h every day in October 2021, over three consecutive days at each of the sampling points. Cow manure is one of the major sources of airborne bacteria (McEachran et al., 2015). Thus, each sample from each type of manure, including fermenting manure (F1), fresh manure (X2), and piled-up manure after fermentation (D3), was collected during the sampling. We simultaneously sampled two replicates at each sampling point, and the samples were incubated at 37 °C for 48 h. These cultivable bacterial colony samples, after incubation, were stored and used for DNA Extraction and 16S rRNA sequencing to analyze and compare air and manure samples in further analysis.

### 2.2 The third-generation sequencing and bioinformatics analysis of microbiome for airborne bacteria

Samples taken from the Coriolis *μ* samplers were transferred into sample tubes, stored in carbon dioxide ice, and sent for DNA extraction and third-generation 16S rRNA sequencing. The total DNA from the samples was extracted using the cetyltrimethylammonium bromide (CTAB)/sodium dodecyl sulfate (SDS) method (van Dijk et al., 2018), and the DNA’s concentration and purity were assessed by DNA agarose gel electrophoresis. The DNA samples were diluted to a concentration of 1 ng/μL. The diluted DNA samples were used for polymerase chain reaction (PCR) with specific probes with barcodes using High-Fidelity PCR Master Mix with GC Buffer Phusion from New England Biolabs. The PCR products were tested and recycled using the QIAGEN toolkit. The extracted and amplified PCR sequence products were used to construct the SMRT Bell library and sent for sequencing using the third-generation sequencing platform from PacBio. The third-generation sequencing platform could obtain the full length of variable sequence regions, while the next-generation sequencing could only obtain one or two variable sequence regions. This largely increases the probability of identifying the correct microbiome species and helps make the composition of microbiomes in the samples clearer. Each sample was sequenced for three replicates. The sequences from the PacBio sequencing platform were then sent for bioinformatics analysis for the next steps, including quality control, sequence alignments, etc. (van Dijk et al., 2018; Zhao et al., 2021).

The raw sequences from PacBio sequencing were in “bam” format, and Lima software was used to differentiate the sample data according to their barcode sequences. Generate Highly Accurate Single-Molecule Consensus Reads (CCS) (SMRT Link version 7.0) (White et al., 2009) was used to trim the sequences with a parameter of CCS = 3 with a minimum accuracy of 0.99 and removing sequences with a length less than 1,340 and the largest length of 1,640. These trimmed sequences were saved as “fastq” and “fasta” formats, and using Simple Sequence Repeat (SSR) and Cutadapt to further cut and remove the probe sequences to remove the sequences with more than eight consecutive bases. After these processes, the final clean reads were kept for further analysis. The raw sequence reads were trimmed to remove the sequence repeats and aligned and mapped to the SSUrRNA reference sequences using the Silva SSUrRNA database version 132^1^ (Pruesse et al., 2007). We used the classification algorithm of “Mothur” in “qiime.ssign_taxonomy” and KRONA (Caporaso et al., 2010) for the visualization of species annotation. The advantage of the third-generation sequencing from the PacBio platform is that the length of sequences is improved, so that the accuracy of the sequencing is also increased, especially for the structure of the sequences (van Dijk et al., 2018; Zhao et al., 2021), which improves the accuracy of microbiome examination using these sequences.

We first annotated these microbiome sequences and clustered the operational taxonomic units (OTUs) from the samples using Uparse software (version 7.0.1001, http://drive5.com/uparse/). This kept the OTUs with the highest appearance frequency and further annotated these OTUs with species using the “Mothur” method (Caporaso et al., 2010) and the SILVA database (see text footnote 1) (Pruesse et al., 2007). We also compare the analysis results with the existing literature. We have been using OTUs in this study, and it was computationally fast and easy for both generation and comparison between samples, and it will generate a more concrete list of microbiomes compared with Amplicon Sequence Variants (ASVs). The OTU approach has served the microbiome community for many years.

The microbiomes were sequenced for 16S gene amplifications in the samples using the third-generation sequencing, that is, the PacBio platform with the SMRT Bell library. These sequences were mapped to the OTU reference sequences and clustered into different microbiome species. According to the clustering of the OTU sequences, the number of sequence reads was counted, standardized, and annotated at the levels of kingdom, phylum, class, order, family, genus, and species. Using MUSCLE (Edgar, 2022) (v3.8.31, http://www.drive5.com/muscle/). The significance of the groups of microbiome abundance in the samples was measured and examined using a *t*-test for two paired groups comparison, a Wilcox test for two unpaired groups comparisons, and multiple testing was applied in the calculation of *p*-values using R. The environmental effects are also correlated with the microbiome’s abundance and are tested for the association significance, which affects the abundance and diversity of the microbiomes.

The alpha and beta diversity were calculated among the OTUs. We further built the phylogenetic tree using the OUT sequences to differentiate the clusters and used PCoA, PCA, and NMDs to reduce the dimensionality and cluster the samples. The alpha diversity, that is, chao1 in the samples, was calculated based on the species abundance in the sample. This alpha diversity describes the diversity in the level of samples and it classifies the individuals based on the microbiome species diversity, that is, there are many species that are only represented by a few individuals in a sample (rare species), compared to the common species, which can be represented by numerous individuals. The beta diversity was calculated based on the method of PCoA, which maps the relative similarities or differences between samples onto a two-dimensional plane for visualization, which basically describes the diversity in the level of microbiomes. We used Qiime software (version 1.9.1) (Caporaso et al., 2010) to calculate “Observed-OTUs, Chao1, Shannon, Simpson, ace, Goods-coverage,” and the PD_whole_tree index, and used R (version 2.15.3) to draw the Rank Abundance curve and make alpha diversity analysis. The PCA analysis was performed using the ade4 and ggplot2 packages, and the PCoA analysis was performed using WGCNA, stats, and ggplot2 packages in R.

### 2.3 Concentration calculation for airborne bacteria and environment monitoring

Samples taken from the six-stage Anderson samplers were transported to the laboratory for incubation at 37 °C for 48–72 h. After incubation, colony numbers were counted and corrected numbers of colonies and the concentration of airborne bacteria were calculated using equations from Ding et al. (2024):


$$\mathrm{Pr}=N\times (\frac{1}{N}+\frac{1}{N-1}+\ldots +\frac{1}{N-r+1})$$

where Pr is the number of colonies after correction, CFU m^−3^; N is the number of sampling holes at each level of the six-stage Anderson sampler; and r is the actual number of colonies, CFU.


$$C=\frac{{\mathrm{Pr}}_{1}+{\mathrm{Pr}}_{2}+{\mathrm{Pr}}_{3}+{\mathrm{Pr}}_{4}+{\mathrm{Pr}}_{5}+{\mathrm{Pr}}_{6}}{t\times F}\times 1,000$$

where C is the concentration of airborne bacteria (in CFU m^−3^); ${\mathrm{Pr}}_{j}$ is the number of colonies after calibration of each sampler stage (where *j* = 1–6); t is the duration of sampling (min); F is the sampling flow rate of the sampler (28.3 L min^−1^). The environment plays an important role in the concentration and microbial composition of airborne bacteria (Quintana et al., 2020). Thus, the microclimate of the dairy farm, including air temperature (T), relative humidity (RH), wind speed (V), wind direction (WD), total irradiance intensity (GHI), ultraviolet irradiance intensity (UV), suspended particulate matter concentration (PM2.5 and PM100), was collected synchronously using a set of environmental monitoring equipment. For more detailed information, see Ru et al. (2023). The parameters including GHI and UV were recorded using a total irradiation meter (MS-40, EKO Corporation, Kyoto, Japan) and a UV irradiation meter (CUV5, K&Z Corporation, Assen, The Netherlands) at a height of 2 m every 30 s and the PM1, PM2.5, PM10, and PM100 were recorded every 5 min using digital universal particle sensors (PMSX003N, Panteng Technology Co., Beijing, China; SDS198, Nuofang Technology Co., Jinan, China).

The association between microbial distribution and environmental factors was tested using canonical correlation analysis (CCA), which can reflect the relationship between microbiome and environmental factors and detect relationships among environmental factors, samples, and microbiomes. It mainly projects points onto vectors with the maximum correlation with the corresponding environmental variables, and it also shows the showing the averages of factor levels. CCA analysis was performed using the “envfit” function in R. CCA projects sites and bacterial communities in the direction of environmental gradients that have the strongest relationship with the bacterial community data, as CCA does not maximize correlation with environmental variables. In CCA, the bacterial community composition is the dependent variable, which is constrained and explained by the environmental variables and sites. The *p*-values were calculated using a permutation test.

## 3 Results

In this section, we aim to analyze the homology between samples and the source of the microorganisms by comparing the microbiome diversity across different farming regions. The sources of microorganisms in the air of cattle farms mainly include the following categories: (1) the background environment; (2) the animal body itself; (3) animal excrement; (4) the products from such as feed and bedding materials; (5) the activities of workers on the farm (Quintana et al., 2020; Zhai et al., 2018; Šantl-Temkiv et al., 2018). Comparing the microbial diversity in feces, background air, office area, and feed processing area can indirectly reflect the source and approximate proportion/importance ranking of air microorganisms enriched in the production area (SN, NT, ZD, and YDC). At the same time, comparing the diversity of microbial components across different production regions and their types (i.e., beneficial and harmful), and analyzing the impact of these components is crucial for the health of living beings, including animals and workers.

### 3.1 Bacterial community abundance and composition

**Figure 1:**
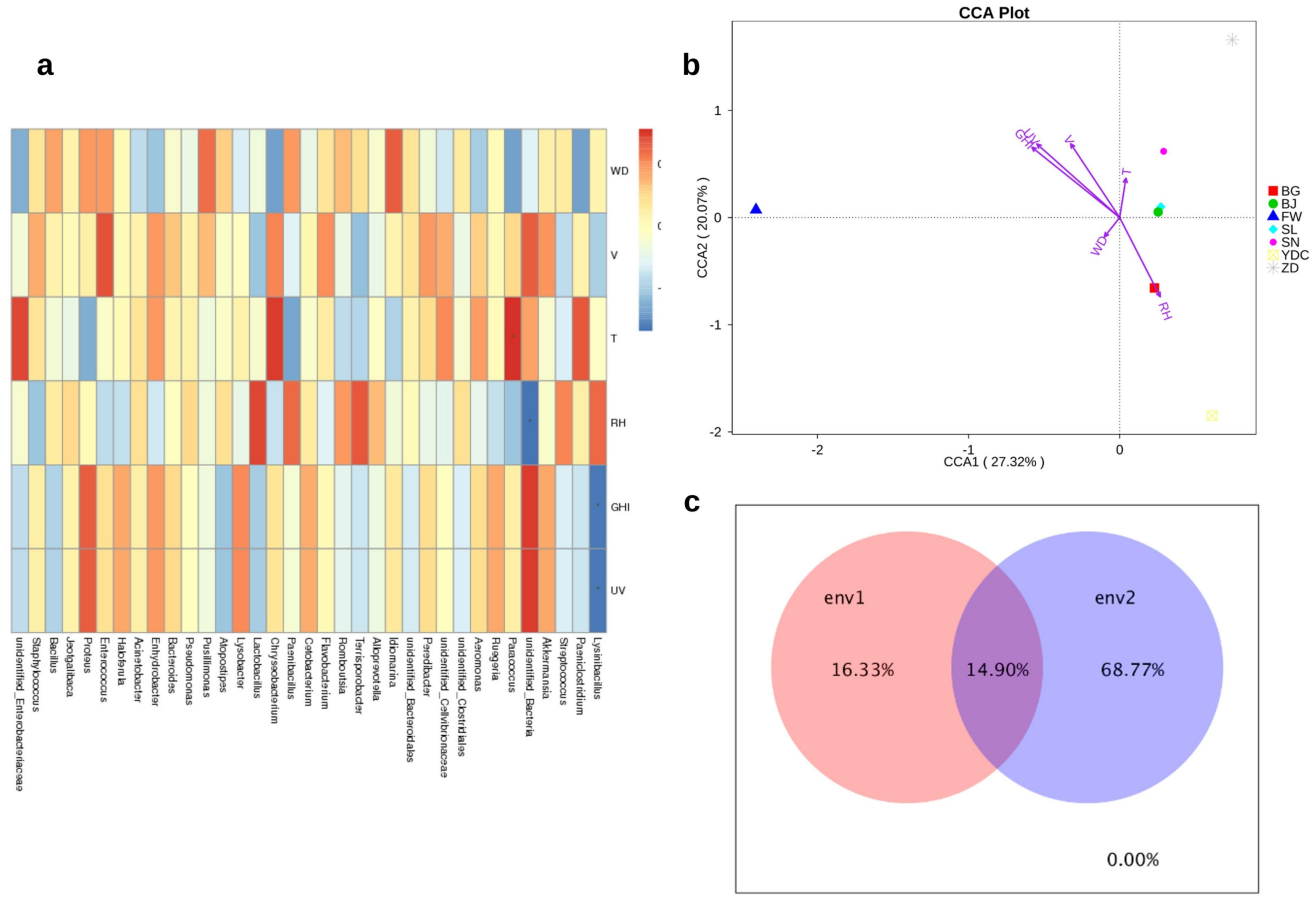
The phylogenetic tree and the relative abundance of the microbiome phyla and genera in the collected samples. **(a)** The phylogenetic tree in the samples; **(b)** The relative abundance of the top 10 genera in the samples; **(c)** The relative abundance of the top 10 genera in the groups.

The final number of raw sequence reads for the microbiomes across the samples is 6,970 on average, ranging from 6,574 to 7,053. The number of clean reads left is. on average, ranging from 6,289 to 6,910 across all the samples. In total, there are 960,7,208 reads called on average, ranging from 9,133,652 to 1,010,7,057 across all the samples. The unique OTUs have been identified in the sequences of these samples. We observed that the highest number of 3,770 unique OTUs was identified in sample “X2” (fresh manure), while the lowest number of 87 unique OTUs was identified in sample “D3” (piled-up manure after fermentation). The phylogenetic tree was built using the microbiome abundance of these samples (White et al., 2009) (Figure 1). It generally reveals the classification of the microbiome phylum and the microbiome diversity of the samples. We have identified phyla of *Proteobacteria*, *Firmicutes*, *Verrucomicrobia*, *Bacteroidetes*, *Fusobacteria*, *Actinobacteria*, *Deinococcus-Thermus*, *Cyanobacteria*, and *Acidobacteria*. We identified that the phyla *Firmicutes* and *Proteobacteria* are mostly enriched in the samples. Among these two phyla, the genus *Staphylococcus*, *Acinetobacter*, *Enterococcus*, and *Bacillus* were found to have the highest abundance in the samples. There were also unknown *Enterobacteriaceae* family enriched in the samples.

### 3.2 Bacterial diversity and variation comparison

To further explore the distribution of the microbiome in the samples, we compared the relative abundance of the microbiome at the genus level in the samples (Figure 1), including *Bacteroides*, *Enhydrobacter*, *Acinetobacter*, *Enterococcus*, *Proteus*, *Jeotgalibaca*, *Bacillus*, and *Staphylococcus*. *Acinetobacter* also appears in 24 of the samples, and *Acinetobacter* species have become increasingly prevalent as causes of nosocomial infections (García-Solache and Rice, 2019; Wisplinghoff et al., 2012). *Staphylococcus* was found in 17 samples and is found on human skin and warm-blooded animals, and is a major cause of wound infections and other infections (Elek and Conen, 1957; Noble, 1998; Serra et al., 2015). *Enterobacteriaceae* was also enriched in 16 samples and is a Gram-negative bacterial family with clinical importance in a variety of infections in humans (van Duin and Doi, 2017).

**Figure 2:**
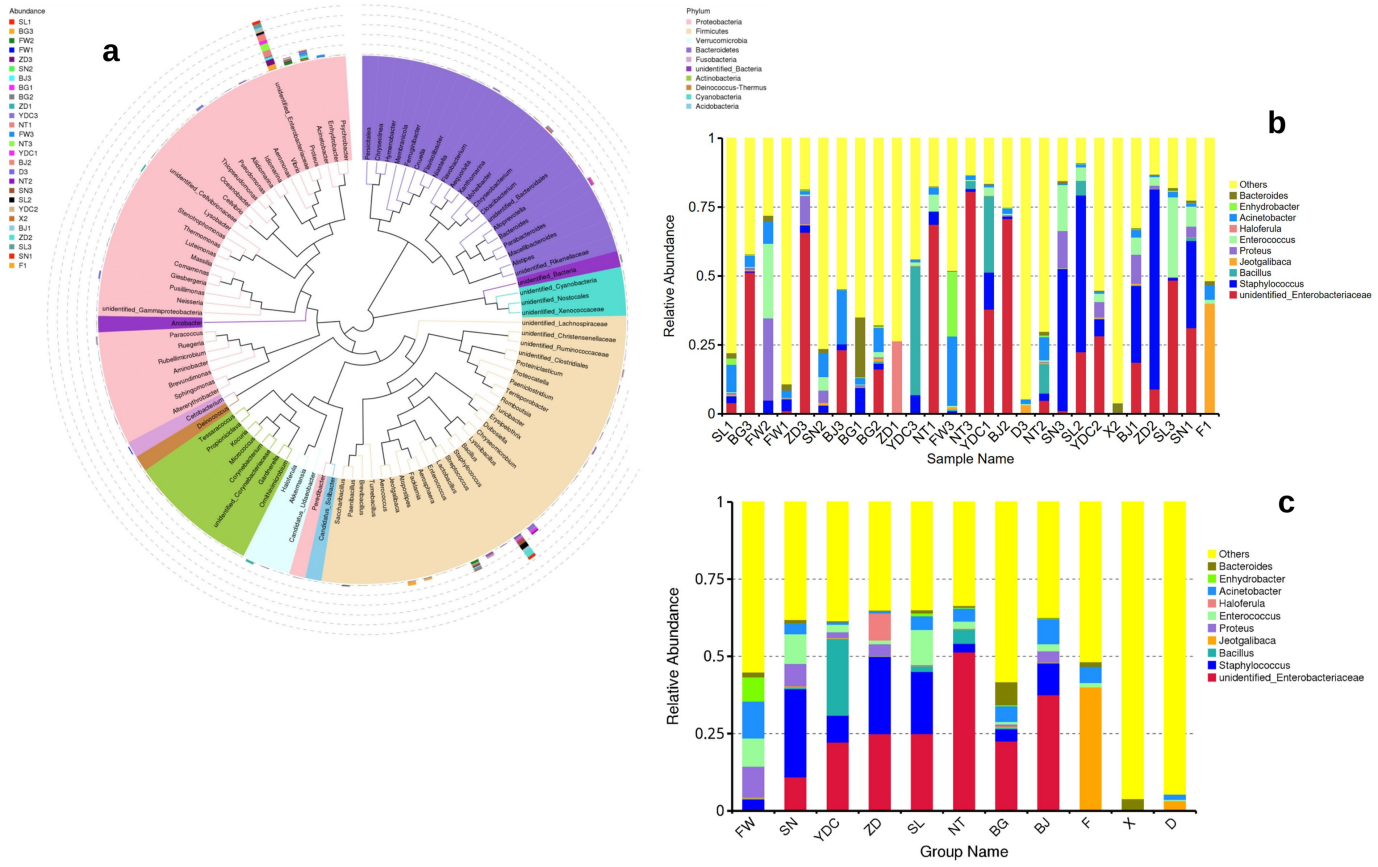
The alpha and beta diversity in the microbiome of the groups: **(a)** The Chao1 alpha diversity in different groups; **(b)** The number of categories of microbiome operational taxonomic units (OTUs) in different groups; **(c)** The beta diversity in the groups using the weighted model.

**Figure 3:**
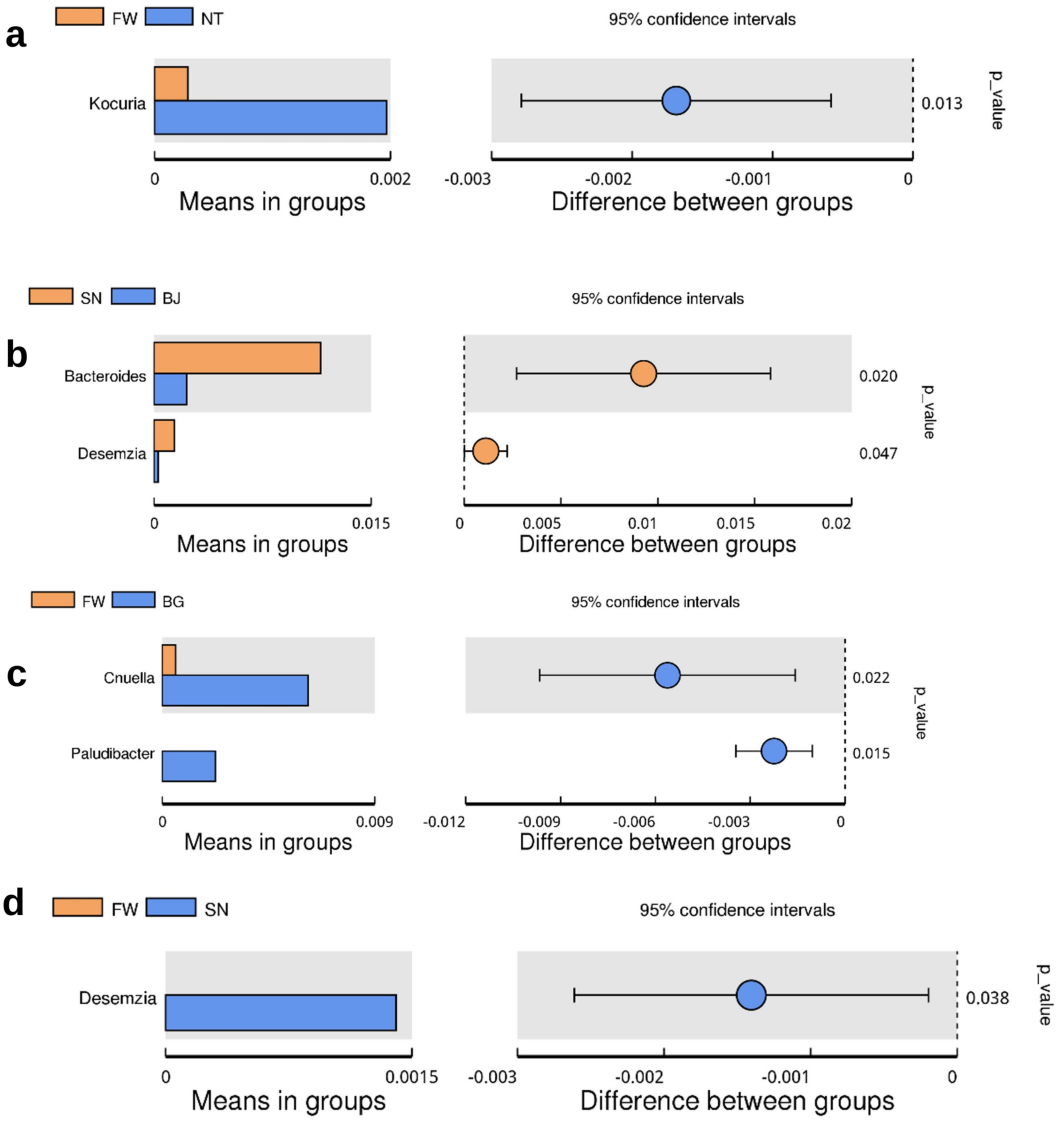
The significance of the comparison of genus means of relative abundance in groups: **(a)** *Kocuria*; **(b)** *Bacteroides* and *Desemzia*; **(c)** *Cnuella* and *Paludibacter*; **(d)** *Desemzia*.

We further compared the microbiome relative abundance in different groups, at the genus level, and it was observed that the genus of *Staphylococcus* and the family of *Enterobacteriaceae* existed in most of the groups except groups fermenting manure (F1), fresh manure (X2), and piled-up manure after fermentation (D3). We next calculated the alpha diversity, that is, chao1, in the samples, which could reflect the diversity at the microbiome level (Figure 2). It was observed that in the air-related grouped samples have relatively higher alpha diversity, while in the manure samples, including fermenting manure (F1), fresh manure (X2), and piled-up manure after fermentation (D3), the alpha diversity is extremely low. The BG group has also relatively lower alpha diversity compared with other air-related grouped samples. Beta diversity, that is, weighted PCoA, was calculated in the samples, and we observed that PC1 explained 32.66% and PC2 explained 22.69% variance in the data. There was no clear clustering among the samples. However, the observed clustering of some samples might be due to the location or the time series when taking the samples. We observed that the relative abundance of the *Kocuria* genus is significantly different between groups FW and NT (*t*-test, *p* < 0.013) (Figure 3).

### 3.3 Environmental factors affecting the bacterial community composition

**Figure 4:**
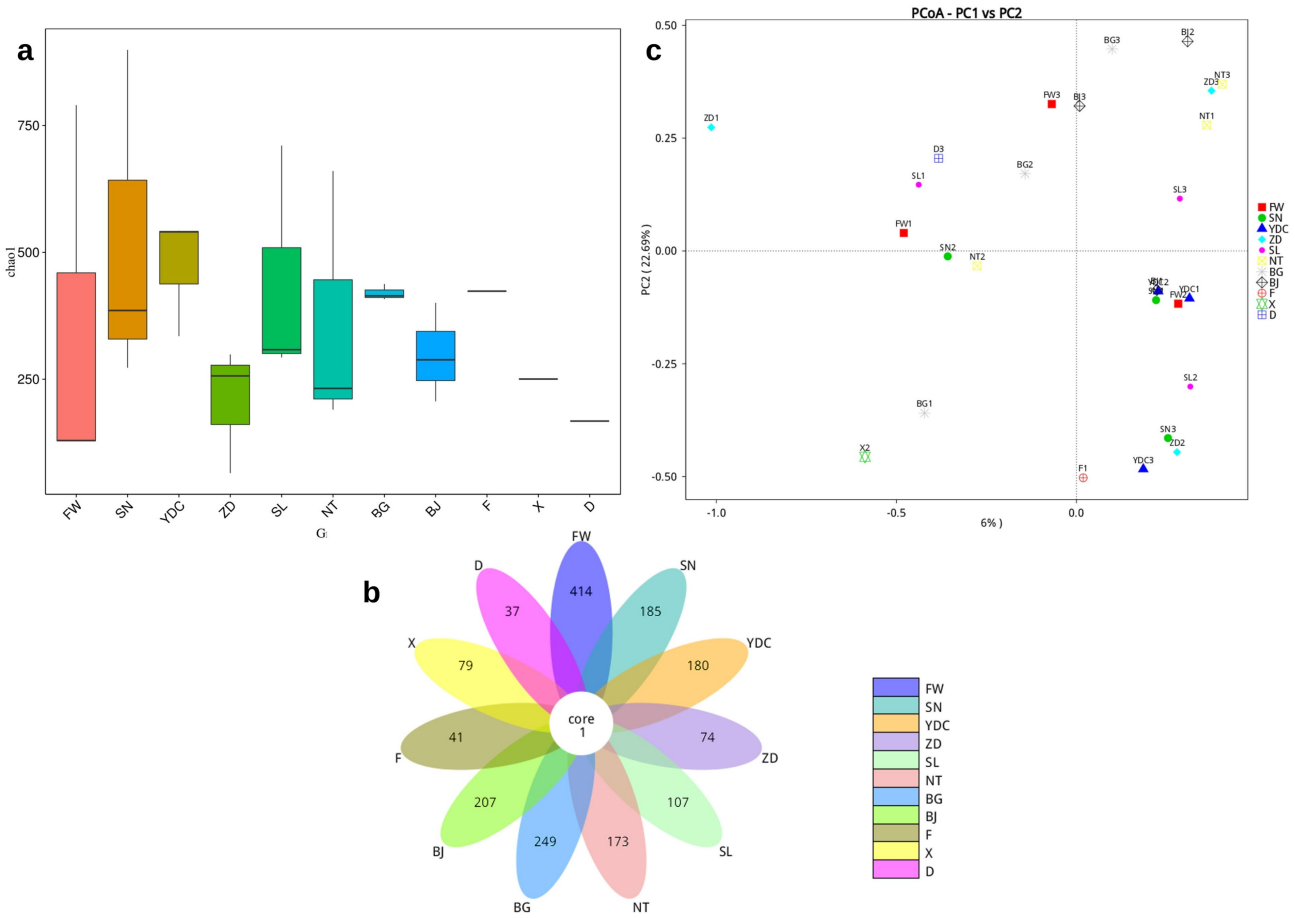
The correlation between environmental factors and the bacterial community composition. **(a)** Heat map of the Spearman correlation analysis between environment and species; **(b)** correlation and canonical correspondence analysis (CCA) plot; **(c)** variance partitioning canonical correspondence analysis (VPA) plot analyzed using one environment factor (i.e., env1: topography factor) and another environment factor (i.e., env2: soil factor).

CCA analysis (Yang et al., 2007) is commonly used to investigate the relationship between microflora and environmental factors and to identify the environmental factors driving the distribution of microbiomes in the samples. Table 1 presents the measured environmental factors at the dairy farm, and Figure 4 shows a heat map of the relationships between these factors and airborne bacterial species. The significance of each environmental factor was tested by the “envfit” function (Ghosh et al., 2015). Figure 4 shows the CCA plot, in which CCA1 and CCA2 refer to the cosine of the angle between the arrow of the environmental factor and the ordering axis, indicating the correlation between the environmental factor and the ordering axis. UV and GHI were the most important influencing factors on species distribution, with R^2^-values of 0.774 (*p* < 0.05) and 0.769 (*p* < 0.05), respectively. WD showed no significant influence on species distribution, with R^2^-values of 0.043 (*p* > 0.05).

**Table 1:** The measured environmental factors in a dairy farm.

T, °C	RH, %	V, m/s	WD, °	GHI, W/m^2^	UV, W/m^2^	PM2.5, μg/m^3^	PM10, μg/m^3^
17.3 ± 2.5	54.5 ± 19.5	0.9 ± 0.7	195.4 ± 116.2	150.4 ± 222.2	7.3 ± 10.5	34.1 ± 29.6	34.9 ± 32.0

RH: relative humidity; WD: wind direction; GHI: global horizontal irradiance; UV: ultraviolet irradiance intensity.

Variance partitioning canonical correspondence analysis (VPA) can reflect the interpretation of each environmental factor on the distribution of microbial communities, and can obtain the contribution degree of each environmental factor that causes the difference in the distribution of microbial communities (Peres-Neto et al., 2006). Based on the counted colonies sampled from the six-stage Andersen samplers, Table 2 shows the calculated concentration of airborne bacteria in different functional zones. We observed that the calculated concentration of airborne bacteria in functional zone DL was the highest, while it was lowest in BG.

**Table 2:** Calculated concentration of airborne bacteria in different functional zones (mean ± SD: CFU/m^3^).

SL	SN	BJ	FW	BG	YDC	DL	NT
3.302 ± 1.552	4.195 ± 1.455	2.248 ± 917	3.064 ± 843	2.184 ± 964	3.780 ± 2.522	4.329 ± 1706	3.476 ± 1.122

SL: feed processing area; SN: dairy barn; BJ: background points; FW: waste management area; BG: office area; YDC: outdoor open lots; DL: production road; NT: milking parlor.

## 4 Discussion

The microbial components of airborne bacteria from different emission sources have specificity, which determines the air quality index in the microclimate of dairy farms and affects the respiratory health and overall immunity of animals and staff (Jahne et al., 2015; Noyes et al., 2016; Zhang et al., 2024). Analyzing the flora structure of air microorganisms in cattle farms can provide a basis for the prevention and control of disease transmission risks and instruct environmental improvement in cattle farms (Jahne et al., 2015). This study analyzed the bacterial composition and abundance of air samples in different functional zones of a cattle farm, including both beneficial and harmful bacterial species.

Among the identified phyla reported in results, it was reported that *Bacteroidetes* is a phylum candidate in targeting infections and antibiotic-associated diarrhea (Ng et al., 2013; Washburne et al., 2018). It was also shown that members of the *Fusobacteria* phylum are associated with the features of important chronic diseases in humans, including colorectal cancer (Bullman et al., 2017). It is notable that the most highly enriched phylum in the samples, *Firmicutes*, plays an important role in chronic diseases, and *Proteobacteria* is a Gram-negative bacterium, responsible for fixing nitrogen, as found by Paterson (2006) and Rizzatti et al. (2017). We also further identified families or classes of bacteria that were enriched in the samples. Lagier et al. showed that *Deinococcus-Thermus* is an anaerobic gram-negative and cancer-promoting bacterium (Lagier et al., 2012; Theodorakopoulos et al., 2013; Weisburg et al., 1989; Finlay et al., 1982) and a potential prognostic biomarker for esophageal cancer (Finlay et al., 1982). It is also notable that *Enterococcus* appears to be in most, that is, 24 out of these samples, which are located in the human gastrointestinal flora and could cause urinary tract infections (Dubin and Pamer Eric, 2017; García-Solache and Rice, 2019; Uttley et al., 1993; Zaheer et al., 2020). The family of unknown *Enterobacteriaceae* existed in most of the groups except the manure samples, suggesting that the air-related samples are highly polluted by these two microbiome genera, with high risk for human infections under expectation (Cho and Blaser, 2012; Jovel et al., 2018; Pflughoeft and Versalovic, 2012).

Among the most highly enriched genus, *Staphylococcus*, could cause various infections, especially wound infections, boils, and skin infections (Navarro Llorens et al., 2010; Papenfort and Bassler, 2016; Poole, 2012). *Enterobacteriaceae* was shown to be a family of gram-negative bacteria that are inhabitants of the normal gut flora and are easily transmitted among patients (Chen et al., 2021; Garrett et al., 2010; Shin et al., 2015; Tong et al., 2015). *Acinetobacter* is a complex genus playing a major role in nosocomial infections found in the literature (Bergogne-Bérézin and Towner, 1996). The genus *Bacillus* could be found mostly in soil, air, water, human and animal gut, and a heterogeneous group, while some species have been identified as opportunistic pathogens or toxin producers in human or animal hosts (Earl et al., 2008). Genus *Kocuria* was found significantly different between the sample groups NT and FW, and Cells of the *Kocuria* genus are Gram-positive cocci that exist in the natural environment and human skin, which is a rare risk bacterium causing infections (Savini et al., 2010). It reveals that in the NT environment, the relative abundance of *Kocuria* is significantly higher than in FW.

In this study, we first reported the bacterial phyla with the found functions from literature we have found in the collected samples, the phylogenetic relationship between the microbiome sequences, alpha and beta diversity of the microbiome, and the abundance of these found microbiome and the difference of abundance between grouped samples (Figures 3, 4). Their potential role in such as zoonotic transmission or antibiotic resistance in the dairy environment, which of these contents are related to the mechanism and pathways of regulating a specific phenotype in these microbiomes, could be our future study directions. However, it should also be noted that there was only a limited sample size, that is, 24 airborne samples and 3 manure samples analyzed in this study. Therefore, more samples could be further collected in future studies to get more statistically meaningful results in terms of the comparisons of compositions of airborne samples.

Environmental factors would affect the reproduction and decay of microbes. We also analyzed the correlations between environmental factors and microbiome species, and between environmental factors and species richness (alpha diversity) to examine the degree of microbial species dependence on the environment and the influence of the environment on community composition. The concentration of airborne bacteria was the highest in DL and lowest in BG, indicating that it was higher in the production area than in the office area. We observed that UV and GHI are highly correlated. GHI is the total irradiance intensity, and UV is the ultraviolet irradiance intensity. We have been measuring GHI in a sun-exposed environment, and we can see that UV is part of GHI. Therefore, we expect UV to be highly correlated with GHI. Generally, the observed trend and enrichment of microbial communities across different functional zones are not as expected.

## 5 Conclusion

This study provides a comprehensive analysis of the airborne bacterial communities in dairy farms, revealing a complex microbiome with significant implications for animal and human health. Our results highlight the presence of both potentially pathogenic and protective bacterial species, emphasizing the dual nature of the microbial environment in livestock farming. The identification of key environmental factors influencing microbial distribution provides valuable insights for developing strategies to manage and improve air quality in these settings. The comparison of bacterial species and abundance across samples and sampling groups suggests that environmental management practices could be leveraged to modulate the microbial balance toward a more protective composition. Future research should focus on the longitudinal monitoring of bacterial communities and the development of targeted interventions to reduce the spread of pathogenic bacteria and enhance the protective effects of the microbiome in livestock farming environments. This will not only contribute to the well-being of farm animals and workers but also have broader implications for public health and the sustainability of agricultural practices.

## Data Availability

All the sequences and related data are deposit in DOI: 10.6084/m9.figshare.28702427.

## Footnotes

1
https://www.arb-silva.de/

## Glossary

OTU: operational taxonomic unit
CCA: canonical correspondence analysis
VPA: variance partitioning analysis
UV: ultraviolet irradiance intensity
GHI: global horizontal irradiance;
CFU: colony forming unit
SD: standard deviation
PCA: principal component analysis
PCoA: principal coordinates analysis
NMDS: non-metric multidimensional scaling;
ASV: amplicon sequence variant
SMRT: single-molecule real-time sequencing
CCS: circular consensus sequencing
SSR: simple sequence repeat
NT: milking parlor
DL: production road
FW: waste management area
BG: office area
SL: feed processing area
SN: dairy barn
YDC: outdoor open lots
IR: interfiled roads
ZD: separation roads
BJ: background points
X: fresh manure
F: fermenting manure
D: piled-up manure after fermentation
RH: relative humidity
WD: wind direction
